# The Role of the ER-Induced UPR Pathway and the Efficacy of Its Inhibitors and Inducers in the Inhibition of Tumor Progression

**DOI:** 10.1155/2019/5729710

**Published:** 2019-02-03

**Authors:** Anna Walczak, Kinga Gradzik, Jacek Kabzinski, Karolina Przybylowska-Sygut, Ireneusz Majsterek

**Affiliations:** Department of Clinical Chemistry and Biochemistry, Medical University of Lodz, Lodz 90-647, Poland

## Abstract

Cancer is the second most frequent cause of death worldwide. It is considered to be one of the most dangerous diseases, and there is still no effective treatment for many types of cancer. Since cancerous cells have a high proliferation rate, it is pivotal for their proper functioning to have the well-functioning protein machinery. Correct protein processing and folding are crucial to maintain tumor homeostasis. Endoplasmic reticulum (ER) stress is one of the leading factors that cause disturbances in these processes. It is induced by impaired function of the ER and accumulation of unfolded proteins. Induction of ER stress affects many molecular pathways that cause the unfolded protein response (UPR). This is the way in which cells can adapt to the new conditions, but when ER stress cannot be resolved, the UPR induces cell death. The molecular mechanisms of this double-edged sword process are involved in the transition of the UPR either in a cell protection mechanism or in apoptosis. However, this process remains poorly understood but seems to be crucial in the treatment of many diseases that are related to ER stress. Hence, understanding the ER stress response, especially in the aspect of pathological consequences of UPR, has the potential to allow us to develop novel therapies and new diagnostic and prognostic markers for cancer.

## 1. Introduction

Cancer refers to any of a large number of diseases characterized by the development of abnormal cells that divide uncontrollably and have the ability to infiltrate and destroy normal body tissue. In the context of rapidly proliferating cells, there is a large demand for protein synthesis [1]. The endoplasmic reticulum (ER) is a cellular organelle responsible for the synthesis and proper folding of transmembrane proteins [2]. Many insults, including hypoxia, nutrient starvation, acidosis, redox imbalance, loss of calcium homeostasis, or exposure to drugs or other compounds, are capable of disturbing ER homeostasis, resulting in diminished capacity for proper protein folding.

These factors can result in unfolded and improperly folded proteins, termed ER stress. Upon ER stress conditions, the activated master regulators of the unfolded protein response (UPR) communicate to the nucleus to regulate the transcription of genes involved in protein folding and processing to increase the ER protein folding capacity, ERAD, and autophagy components. This further leads to reduction in ER workload and cell survival and death factors to determine the fate of the cell depending on the ER stress condition [3]. Cancerous cells rely on these UPR pathways to adapt to perturbations in ER folding capacity due to the hostile tumor microenvironment as well as the increase in unfolded and misfolded proteins [4]. When the UPR fails to restore ER homeostasis and attenuate ER stress, the UPR activation induces apoptosis [5]. Therefore, UPR can be therapeutically exploited to reduce the survivability of malignant cells or tip the balance towards apoptosis.

In this review, we have discussed the studies on ER stress-induced UPR signaling in cancer as well as other various diseases and applications of ER stress-modulating molecules in therapy. The use of PERK kinase inhibitors appears to be a chance for a modern personalized therapy for people for whom other therapies have failed. This article is a short analysis of publications published so far in this field.

## 2. ER Stress, UPR, and Their Role in the Disease Development

The stress of the endoplasmic reticulum (ER) can be induced by various factors. In response to it, the UPR pathway is activated. It is responsible for preservation of cell homeostasis. This ER balance can be perturbed by physiological and pathological insults such as high protein demand, infections, environmental toxins, inflammatory cytokines, and mutant protein expression resulting in the accumulation of misfolded and unfolded proteins in the ER lumen, a condition termed as ER stress.

The stress of the endoplasmic reticulum is associated with the activation of three factors: PKR-like ER kinase (PERK), activating transcription factor 6 (ATF6), and inositol-requiring enzyme 1 (IRE1*α*). Studies on the role of this pathway and the effects of its inhibition show different results depending on the activated factor and the type of cancer.

The regulation of the protein synthesis process in response to stress conditions is based on the phosphorylation of the eIF2*α* factor by PERK kinase [6]. Interestingly, higher levels of the phosphorylated eIF2*α* protein have been discovered in the course of neoplastic diseases, e.g., breast cancer [7]. Activation of the UPR pathway results in the restoration of cellular homeostasis by increasing the translation of ATF4 mRNA which is responsible for the expression of proadaptive genes needed to transmit a signal that allows the cell to survive during stressful conditions [8]. The prolonged stress of the endoplasmic reticulum results in an increased transcription of the CCAAT-enhancer-binding protein homologous (CHOP) protein [9]. It is a factor that can both direct the cell to the pathway of programmed death (by weakening the expression of antiproapoptotic Bcl-2 proteins and activation of BIM proteins that bring cells to the apoptosis pathway and enable cell survival by inducing the expression of the GADD34 and ERO1*α* genes [6, 10]. On the other hand, it is responsible for the weakening of the UPR associated with PERK kinase and the proapoptotic response induced by the CHOP protein [11, 12].

Other pathway that partially has a crosstalk with the PERK branch of UPR is IRE1*α*. IRE1*α* is a kinase that undergoes autotransphosphorylation upon ER stress conditions, leading to endoRNase activation. Active IRE1 introduces nicks in X-box-binding protein-1 (XBP1) mRNA, and ligation of the remaining 5' and 3' fragments resulting in the activation of XBP1s (spliced form) Lu et al. [13]. It modulates the expression of several UPR target genes involved in ER folding, glycosylation, and ER-associated degradation (ERAD) [14]. Moreover, the IRE1/endo-RNAse activity can affect mRNAs and microRNAs and cause regulated IRE1-dependent decay (RIDD). RIDD has emerged as a novel UPR regulatory component that controls cell fate under ER stress [15].

**Figure 1:**
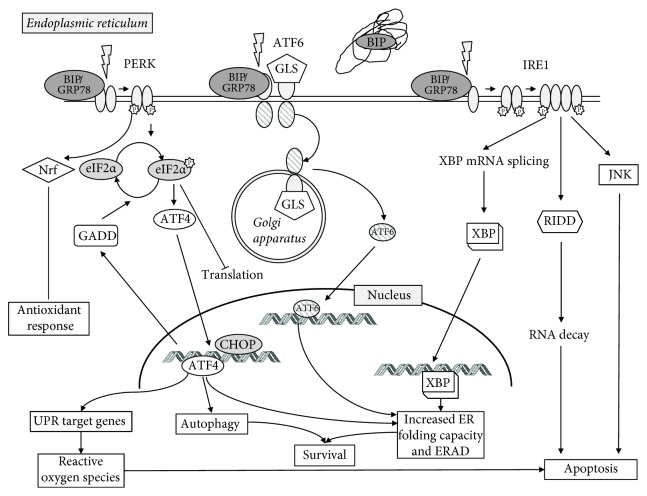
The UPR signaling cascade. UPR pathways are activated through competitive binding of the chaperone immunoglobulin heavy-chain-binding protein (BiP) also known as glucose-regulated protein 78 (GRP78) to the receptors. Accumulation of misfolded or unfolded proteins in the endoplasmic reticulum (ER) leads to the dissociation of BiP from 3 transducers: PERK (double-stranded RNA-activated protein kinase-like ER kinase), ATF6 (activating transcription factor 6), and IRE1 (inositol-requiring enzyme). Upon activation, PERK phosphorylates and deactivates the eukaryotic initiation factor (eIF2*α*), which results in an increased level of ATF4. This triggers the activation of C/EBP homologous protein (CHOP). Subsequently, DNA damage-inducible protein transcript (GADD) expression is also elevated, what negatively regulates eIF2*α* phosphorylation and restores translation. While initially contributing to cellular survival in conditions of ER stress, PERK is considered proapoptotic due to strong induction of CHOP in chronic or terminal ER stress. PERK also regulates several transcription factors including NRF-2 that upregulate the antioxidant response and ATF4 which can lead to both protective and apoptotic signaling. Upon activation, ATF6 is released from BiP that is trafficked to the Golgi apparatus (it consists of two Golgi localization signals, GLS) and cleaved by the proteases into two subunits. Then it translocates to the nucleus where it is the promoter region of UPR target genes termed the endoplasmic reticulum stress element (ERSE), activating genes responsible for the components of the UPR response and leads to the induction of molecular chaperones (e.g., GRP78, Grp94, and calreticulin, as well as CHOP and XBP1). The various ER chaperones are part of a protective adaptive response that regulates protein folding and other components of the UPR. ATF6 is primarily considered prosurvival due to its role in promoting the transcription of chaperones and XBP1. IRE-1 activation is responsible for in the unconventional splicing of XBP-1 mRNA. Spliced XBP-1 encodes a transcription factor that activates the expression of UPR genes, such as chaperones and ER-associated degradation proteins (ERAD). These include the activation of the cell death machinery, degradation of ER-localized mRNAs that encode secreted and membrane proteins through the RIDD (regulated Ire1-dependent decay) pathway, and induction of autophagosomes. This signaling cascade increases the folding capacity of the ER and causes degradation of misfolded proteins. IRE1 is mainly considered as a prosurvival pathway, but it also can contribute to apoptosis through the activation of JNK-dependent pathway.

The last branch of ER stress-induced cellular response via UPR is the activation of ATF6. It was primarily identified as a cytoprotective factor during ER stress [16]. ATF6 is activated by proteolysis and acts as a transcriptional factor for regulating the downstream expression of genes responsible for stresses [17]. Studies have shown that activated ATF6 signaling is correlated with lower OS of patients with various types of tumors, cancer recurrence, metastatic lesions, tumor growth, and resistance to radio- and chemotherapy [18]. The UPR signaling cascade is shown in Figure 1.

## 3. Major Inducers of ER Stress

UPR is a factor of known prosurvival factor of tumor cells that can act via adaptive mechanism during cancer progression. In the context of cancer, different extrinsic (hypoxia, nutrient deprivation, and acidosis) and intrinsic (oncogene activation) factors cause endoplasmic reticulum stress and trigger the UPR.

One of the major factors inducing the UPR pathway is hypoxia. The tumor microenvironment is characterized by low oxygen concentration that is related to rapid tumor growth. Cancer cells in this environment show a high proliferative potential and, together with the increase in oxygen concentration, an increasingly aggressive phenotype. Previous studies suggest that hypoxia weakens protein biosynthesis due to the stress of the endoplasmic reticulum, which leads to the activation of the response pathway to UPR. Activation of the UPR in hypoxic tumors leads to increased autophagy [19]. Autophagy liberates amino acids from long-lived proteins and damaged organelles. In multiple cell lines, PERK mediates the upregulation of LC3 and autophagy-related gene 5 via ATF4 and CHOP, respectively, promoting phagophore formation.

Oxidative stress is also one of the main factors causing ER stress. Reactive oxygen species (ROS), i.e., molecules having an unpaired electron, such as hydroxyl (OH) and superoxide (O_2_) radicals, are formed endogenously during the processes occurring in the respiratory chain in the mitochondria; hence, their increased amount can be observed in cells with high-energy demand. O_2_ may form nitrate (ONOO-) together with nitric oxide (NO), which is an extremely overreactive molecule and may interfere with proteins and DNA causing their oxidation or nitration [20]. They arise in large quantities under hypoxia conditions, which stimulate mitochondrial activity. Free radicals can also be delivered to the body exogenously by eating fried and grilled products. Their production is also induced by smoking cigarettes. Free radicals in the human body perform many roles such as signaling, regulation of gene expression, or modulating the level of calcium in the cell [20]. Their excess, however, can be harmful. Oxidative stress interferes with the process of protein folding, leading to the formation of deposits of unfolded proteins, which induces ER stress [21]. Studies carried out on mice may confirm this directly [22]. In transgenic animals that overexpressed the superoxide dismutase (SOD) gene, ATF4 and CHOP levels were observed to be lower than in wild type. It follows that the apoptotic death of hippocampal cells after ischemia associated with ER stress in these mice occurs to a lesser extent if the process of eliminating free radicals is more efficient.

Induction of oxidative stress is closely related to inflammatory processes. Chronic inflammation can lead to the release of inflammatory factors such as prostaglandins, production of ROS, and secretion of tumor-promoting cytokines. These molecules promote the survival, growth, and metastasis of tumor cells through NFKB/NFkB (nuclear factor kappa B; mediators downstream of the UPR), STAT3 (signal transducer and activator of transcription 3), and AP-1 (AP-1 transcription factor) signaling pathways as well as cytokines such as IL1B/IL1b, IL6, IL11, and IL23A [23]. Experiments performed on pancreatic islet cells and in mice with type 2 diabetes mellitus showed that cytokines such as IL-1B, IL-23, and IL-24 can induce ER stress [21]. By administering serum with antibodies against this particular interleukin, an improved glycemic control and a reduction in ER stress were achieved. The experiments carried out in 2010 by scientists from Belgium, Germany, Greece, and USA have also shown that interferons can cause disturbances leading to excessive ER stress [24].

Other factor that can induce ER stress is ionizing radiation (IR). It is proven that IR can evoke the activation of the PERK-eIF2*α* pathway and subsequently cell death [25, 26].

During cancer genesis, an acute demand of protein synthesis is also needed to support different cellular functions, such as tumor proliferation, migration, and differentiation, often driven by oncogenic activation [27]. Eukaryotic cells react to the nutrient starvation by activation of the integrated stress response (ISR). It is driven by kinases (including GCN2 and PERK kinase) that induce eIF2*α* phosphorylation and translation of ATF4 [28]. ATF4 regulates adaptation to amino acid deprivation (AAD) by regulation of amino acid transporter expression (SLC3A2, SLC7A5, and GLYT1) and enzymes of amino acids metabolism. Additionally, activation of ATF4 is also vital for suppressing oxidative stress through the induction of glutathione biosynthesis [29]. ATF4 is a protein necessary for cancer cells growth proliferation. Data has shown that ATF4-deficient cell cultures have to be supplemented with antioxidants and necessary amino acids to survive [30, 31]. GCN2 activation/overexpression and increased phospho-eIF2*α* were observed in human and mouse tumors compared with normal tissues and abrogation of ATF4 or GCN2 expression significantly inhibited tumor growth *in vivo* [31]. Additionally, Wang et al. [32] showed that amino acid deprivation promotes tumor angiogenesis through the GCN2/ATF4 pathway [32].

UPR can also be induced by glucose deprivation and subsequent acidosis. Tumor cells adapt to low glucose levels by switching to a high rate of aerobic glycolysis, which is correlated with the expression of glucose transporter GLUT1 [33]. The resulting lactic acid production reduces the pH and thus causes acidosis. It is an important feature of the tumor microenvironment that can increase tumor survival rate and its progression by the regulation of CHOP and BCL-2 (B-cell leukemia/lymphoma-2) protein family members [34]. The glucose-regulated protein family, which includes the master UPR regulator GRP78, was discovered due to the upregulation of its members in response to glucose deprivation [35]. Also, elevated XBP1 splicing level was observed upon exposure to a nonmetabolizable glucose analog that simulates glucose deprivation [36].

The most potent intrinsic factors that induce UPR are activated oncogenes. We will discuss three of them: RAS, BRAF, and c-MYC.

Data show that oncogenic HRAS induces and activates the IRE1*α* RNase in primary epidermal keratinocytes through the MEK-ERK pathway and that IRE1*α* and *Xbp1* splicing are elevated in mouse cutaneous squamous tumors [37]. Moreover, HRAS(G12V)-driven senescence was mediated by the activation of all arms the ER-associated unfolded protein response. It was also found that oncogenic forms of HRAS (HRAS(G12V)), but not its downstream target BRAF (BRAF(V600E)), engaged a rapid cell-cycle arrest and were associated with massive vacuolization and expansion of the ER [38]. ATF4-deficient MEFs transformed with SV40 large T antigen and HRAS(G12V) oncogenes displayed a slow growth, failed to form colonies on soft agar, and formed significantly smaller tumors in vivo due to suppressing expression of the INK4a/ARF [39]. Transformation of PERK-deficient cells by SV40 large T antigen and K-RAS (G12V) did not affect growth and anchorage-independent growth, suggesting that ATF4 could have some PERK-independent functions during transformation [40]. Increased levels of p-eIF2*α*, XPB1s, and GRP78 were observed in Nf1/p53 mutant mouse model of malignant peripheral nerve sheath tumors (MPNSTs), suggesting that the UPR is activated in HRAS-driven tumors *in vivo* [41].

The BRAF(V600E) mutation is present in up to 70% of malignant melanoma and other cancers and results in an increased activation of the kinase, leading to enhanced MEK/ERK signaling in the absence of extracellular signals [42]. It was proven that the presence of this mutation increased protein synthesis and activated XBP1 and GRP78 in human melanocytes. Activation of the UPR was dependent on protein synthesis, as suppression of protein synthesis attenuates the activation of XBP1s and GRP78 as well as induced autophagy via IRE1 and PERK [43–45].

c-Myc drives important biological processes such as cell growth, proliferation, and its metabolism (especially protein synthesis) and regulates apoptosis [46]. Recent studies showed that cell autonomous stress, such as activation of the protooncogene *MYC/c-Myc*, can also trigger the UPR. It was demonstrated that c-Myc and N-Myc activated the PERK/eIF2*α*/ATF4 arm of the UPR, leading to an increased cell survival via the induction of cytoprotective autophagy. Inhibition of PERK significantly reduced Myc-induced autophagy, colony formation, and tumor formation. Moreover, pharmacologic or genetic inhibition of autophagy resulted in increased Myc-dependent apoptosis [47]. Dey et al. [48] also observed EIF2AK3/PERK-dependent induction of cytoprotective autophagy in MYC-overexpressing cells. The deregulated expression of Myc drives tumor progression in most human cancers, and UPR and autophagy have been implicated in the survival of Myc-dependent cancer cells. Data obtained in the animal model (*Drosophila melanogaster*) show that UPR, autophagy, and p62/Nrf2 signaling are required for Myc-dependent cell growth [49].

A number of studies confirm the role of the excess of unfolded proteins in the induction of the PERK kinase-dependent pathway. ER stress is induced to restore cell homeostasis by inhibiting translation.

## 4. Cancer Cell Targeting via Apoptosis Pathway or Promoting Cell Survival

The stress of the endoplasmic reticulum is associated with the activation of three factors: PERK, ATF6, and IRE1a. Studies on the role of this pathway and the effects of its inhibition show different results depending on the activated factor and the type of cancer.

The role of ER stress as an important factor in cancer development has been proposed in 2004, and since then there are more and more evidence confirming this thesis [50]. For instance, increased expression levels of the major components of the UPR such as PERK and ATF6, IRE1*α*, both unspliced and spliced XBP1, were observed in tissue sections from a variety of human tumors including brain, breast, gastric, kidney, liver, lung, and pancreatic cancers [51–58]. Moreover, the chaperone GRP78 that is linked to higher tumor grades dissemination/metastasis of human tumors and reduced overall survival (OS).

Rubio-Patino et al. [59] showed that in mice with colorectal malignancies, activation of the IRE1-associated UPR pathway led to reduced tumor growth and increased survival [59]. This study, through a low-protein diet, induced ER stress in tumor cells. During the experiment, it also turned out that under such conditions the immune response is much more efficient; these mice had an increased number of NK cells and CD3 + CD8 + lymphocytes infiltrating the tumor. Inhibition of this pathway by the inhibitor resulted in a reduction in the beneficial effect of the low-protein diet, which suggests that the UPR-related pathway associated with IRE1a directed the cells to the pathway of apoptosis and increased sensitivity to the immune system.

It should be noted that studies regarding the role of IRE1a activated in the group of patients with breast cancer showed that splicing XBP1 associated with the above-mentioned factor leads to the adaptation of cells to the conditions of hypoxia [60]. Such tumors are characterized by a worse prognosis. This underlines the very important role of accurate determination of the impact of UPR pathway activation on tumor progression.

In patients with chronic B-chronic lymphocytic leukemia (B-CLL), it was shown that the induction of the UPR pathway associated with ER stress (activation of PERK kinase) leads to apoptotic death of tumor cells. This effect was confirmed by the influence of commercially available ER stress inducers (thapsigargin and tunicamycin) on the progression of tumor growth. Researchers have shown that these compounds induce apoptosis of cells in patients with B-CLL [61]. On the other hand, ER stress also triggers survival signals in B-CLL cells by increasing BiP/GRP78 expression.

The branch of the UPR pathway associated with PERK kinase is responsible for the induction of blood vessel formation in tumor cells under hypoxic conditions. Angiogenesis is mediated by ATF4, which induces the expression of vascular endothelial growth factor (VEGF) [62]. Data have shown that ATF4 binds to the regulatory site of VEGF [63]. Moreover, *in vitro* studies revealed that partially blocking UPR signaling by silencing PERK or ATF4 significantly reduced the production of angiogenesis mediators induced by glucose deprivation [63].

In the melanoma patients, the role of the UPR pathway in promoting cell survival has been confirmed [64]. It induces the expression of proadaptive proteins and at the same time lowers proapoptotic proteins. It also increases the process of autophagy, which allows cancer cells to recover the necessary components, such as amino acids, and remove damaged organelles from cells that are older and more damaged.

It has also been confirmed that the UPR pathway associated with PERK promotes the progression of colorectal tumors. It has been shown that PERK plays an important role in tumor cell adaptation to hypoxic stress by regulating the translation of molecules that promotes cellular adhesion, integrin binding, and capillary morphogenesis necessary for the development of functional microvessels [65]. The association of ATF4 factor promoting angiogenesis and proadaptive gene expression is suspected, and GADD34, which prevents apoptosis induction during prolonged ER stress, by lowering overtranslation of proteins [66].

Pancreatic cancer cells are under permanent high hypoxic state caused by large volume of the tumor, and only a small fraction of cancer cells are at the normal oxygenation levels of the surrounding normal pancreas [67]. Choe et al. (2011) showed that in pancreatic cancer cells, activation of the PERK and IRE1 arms of the UPR are delayed in the presence of ER stressors, compared to normal pancreatic cells. This was attributed to an abundance of protein-folding machinery, such as chaperones. Additionally, once activated, the prosurvival XBP1 was noted to be activated for a longer period of time in cancer cells when compared to normal cells [68]. Moreover, the unfolded protein response seems to play a predominant homeostatic role in response to mitochondrial stress in pancreatic stellate cells. Su et al. evaluated AMPK/mTOR signaling, autophagy, and the UPR to cell fate responses during metabolic stress induced by mitochondrial dysfunction [69]. Rottlerin treatment induced rapid and sustained PERK/CHOP UPR signaling, causing loss of cell viability and cell death. As well as adapting to chronic ER stress, it has been recently postulated that anterior-gradient 2 (AGR2) may contribute to the initiation and development of PDAC [70].

In addition, the experiment conducted by Liu et al. [71] showed that activation of the UPR pathway leads to the change in ATF6*α*, PERK, and IRE1*α* expression and is associated with progression of prostate cancer, worse prognosis, and more aggressive growth [71].

The summary and additional information of the UPR involvement in the pathogenesis and progression of various types of cancer is presented in Table 1.

## 5. ER Stress and Cancer Treatment—Novel UPR Modulating Factors

ER stress plays a large role in both progression and moderation of response to cancer chemo- and radiotherapy. Activation of the UPR pathway takes place under the influence of many factors, which are subjected to a cancer cell: unfolded proteins (protein economy is intensified during cancer, which is a very dynamic process), hypoxia (associated with excessively fast nascent tumor mass), pH changes, or chemotherapy [84].

GPR78 as the chaperone protein is an interesting target for the anticancer therapy, especially in cancer steam cells, and was partially effective in head and neck cancer treatment [85]. An immune adjuvant therapy seems to be effective since monoclonal antibody against GRP78 was shown to suppress signaling through the PI3K/Akt/mTOR pathway, which is responsible for radiation resistance in nonsmall cell lung cancer and glioblastoma multiforme (GBM). It was shown that ionizing radiation increased GRP78 expression through the induction of ER stress, and treatment with the monoclonal antibody along with ionizing radiation in mouse xenograph models showed a significant tumor growth delay [86]. Other study reveals that using a phage, displaying a ligand specific to GRP78 with the antiviral drug ganciclovir, prostate cancer bone metastasis tumors were reduced by an average of 50% [87].

A group of patients with AML has been studied for molecular changes that allow survival and resistance to treatment. The results clearly indicate the role of the proadaptive pathway associated with ER stress mediated by PERK kinase. In the case of PERK, selective ATP-competitive *PERK* kinase inhibitors such as GSK2606414 or GSK2656157 were antiproliferative in multiple cancer models in vivo including multiple myeloma [88, 89]. In the AML cells obtained from the mouse model in which GSK2656157, a PERK inhibitor, was used, the response to treatment was better. An 80% greater decrease in tumor colony growth was obtained against the group in which the UPR pathway occurred correctly [90]. In case of human multiple myeloma, other ER stress modulator STF-083010, a small-molecule inhibitor of Ire1, is a promising target for anticancer therapy [91].

It has been demonstrated that tyrosine kinase inhibitors (TKIs) on Hodgkin's lymphoma are correlated to increase in ER stress and ER stress-induced apoptosis. After treatment of L-428, L-1236, and KM-H2 cells with the TKI sorafenib, the elevated level of p-PERK and phosphorylation of eIF2*α* were observed. In addition, proapoptotic signaling molecules GADD34 and CHOP were noted to be upregulated after incubation with sorafenib [92].

It has been also proven that PERK regulates glioblastoma sensitivity to ER stress through promoting radiation resistance [25]. By inhibiting PERK, it was determined that ionizing radiation- (IR-) induced PERK activity led to eIF2*α* phosphorylation. IR enhanced the prodeath component of PERK signaling in cells treated with Sal003, an inhibitor of phospho-eIF2*α* phosphatase. Mechanistically, ATF4 mediated the prosurvival activity during the radiation response. The data support the notion that induction of ER stress signaling by radiation contributes to adaptive survival mechanisms during radiotherapy.

Adaptation to an environment conducive to ER stress is essential for survival and propagation of pancreatic cancer cells. In vitro studies of diindolylmethane derivatives have shown similar ER stress induction activity as thapsigargin followed by subsequent apoptosis via death receptor 5 (DR5) through induction by CHOP [93]. Other compound, a proteasome inhibitor called bortezomib, was increasing the levels of GRP78, CHOP, and c-Jun NH2 terminal kinase (JNK) in L3.6pl pancreatic cancer cells, yet interestingly at the same time was blocking PERK autophosphorylation, and thus inhibiting phosphorylation of eIF2*α* [94].

ER stress can be a factor supporting the progression of colorectal cancer. It has been proven that in cell lines of colorectal cancer it plays an important role in the loss of the intestinal stem cell (ISC) phenotype. Activation of the PERK eIF2*α* branch in response to ER stress leads to the transformation of CRC cells to a more aggressive type [84].

It has been shown that activation of the UPR pathway and adaptation to stress conditions lead to the emergence of a chemotherapy-resistant phenotype HT-29/MDR [95]. This process takes place by activating the PERK/Nrf2/MRP1 axis. MRP1 is a protein belonging to membrane transporters. Its activity is inversely proportional to the concentration of doxorubicin in the cell. The induction of MRP1 protein expression by PERK kinase under ER stress conditions was associated with a lower concentration of the chemotherapeutic agent in the cell and hence resistance to treatment [95].

Activation of the UPR pathway in response to ER stress involves targeting the cell both to the apoptosis pathway and to enable its survival. The pathways leading to cell survival allow clones to resist both treatment [84] and those more susceptible depending on the type of chemotherapy and tumor phenotype used [96].

The research conducted by Wielenga et al. [96] in colorectal cancer cells showed that the induction of tumor cell differentiation before stress ER leads to the formation of clones that are more susceptible to chemotherapy [96]. Cell lines taken from patients with colorectal cancer were exposed to an ER stress inducer (subtilase cytotoxin AB, SubAB). The results were as follows: in vitro activation of the UPR pathway led to the differentiation of tumor cells whose colonies had increased sensitivity to chemotherapy in the form of oxaliplatin. In vivo, supportive treatment in the form of SubAB was shown to improve the tumor response to oxaliplatin, but the experiment did not prove in this model that this was directly due to the changes in the phenotype of the derived cells.

The induction of ER stress with various substances, moderating the course, blocking the branches of the UPR pathway is currently used in *in vitro* and *in vivo* models to assess their impact on growth and progression of CRC. Treatment trials are divided into two streams of ER stress use. One of them induces it with compounds that activate the proapototic pathway. The other uses the assumption that CRC stem cells, thanks to the PERK/eIF2*α* pathway, differentiate into more aggressive phenotypes and the fact that the primary role of the UPR pathway is to restore homeostasis in the cell and allow it to survive under stress conditions through its other branches.

Yang et al. [97] using levistolide A induced the formation of free radicals that caused ER stress [97]. The wild type and p53-/- CRC colonies treated with this compound were reduced, since the cells were subject to apoptosis. Administration of N-acetylcysteine, which blocked the action of levistolide A, had an effect in the reduction of tumor mass.

The effect of tolfenamic acid, which belongs to the NSAIDs group, was also investigated on the development of CRC [98]. Tolfenamic acid promotes ER stress, resulting in the activation of the unfolded UPR signaling pathway, of which PERK-mediated phosphorylation of eukaryotic translation initiation factor 2*α* (eIF2*α*) induces the repression of cyclin D1 translation. In mice with FAP syndrome, the apoptosis in CRC cells was induced through the branch associated with ATF4. It also correlated positively with the decrease in the concentration of cyclin D1 and the activity of Rb oncogene. The result of this study may suggest a likely mechanism of beneficial effects of NSAIDs on the risk of CRC.

Other study confirms the positive effect on CRC tumor regression, due to the activation of the branches associated with CHOP, Bax, and caspase 3 in andrographolide therapies [99]. This compound increases the production of free radicals and induces ER stress, which leads cells to the path of apoptosis. In addition, decreased concentrations of cyclins have also been demonstrated, which in turn inhibits the progression of the cell cycle.

Studies are not limited to the UPR modulators mentioned above. Since it is a very promising target for novel anticancer therapy, more and more new molecules are being tested. A significant amount of them are naturally occurring chemicals that are present also in plants. Due to the abundance of the compounds affecting UPR in Table 2, we have summarized the literature review on tested modulators in various cancer cell lines.

## 6. Conclusion

Stress of the endoplasmic reticulum is a process commonly occurring under the influence of various factors (free radicals, unfolded or misfolded proteins). The UPR pathway is the physiological response of the cell to the stress conditions affecting the cell. ER stress response has been highlighted as a key factor (next to the mutations) occurring at various stages of the disease progression and the individual response to the treatment. Cancers are a very heterogeneous group in which the UPR pathway can lead to adaptation to stress conditions (e.g., hypoxia in rapidly growing tumors), apoptosis (strengthening the immune response in colorectal cancer cells or induction of apoptosis in B-CLL cells). At the same time, depending on the circumstances and cell's condition, it can lead to resistance to treatment and production of clones less sensitive to chemotherapy. UPR activation is a vital step for oncogenic transformation, as UPR signaling molecules interact with well-established oncogene and tumor suppressor gene networks to modulate their function during cancer development.

UPR modulators are a promising hope for a personalized therapy for patients in whom chemotherapy or radiotherapy have failed. It can become an innovative way to fight several different types of cancer. The response to a given compound depends on the phenotype of tumor cells, the severity of the disease, and the chemotherapy used so far.

It is emphasized that further experiments and analyses should be carried out using a variety of compounds that have the ability to inhibit and induce the UPR pathway in different types of cancers. It could also be useful in the treatment of noncancerous diseases.

## Figures and Tables

**Table 1:** UPR involvement in cancers.

UPR linked to cancer	Cancer type	Branch of the UPR	References
Cancer initiation	CRC	PERK/eIF2*α* axis activation is associated with the loss of stemness IRE1*α* pathway induces intestinal stem cell expansion	[72, 73]
	Colitis-associated cancer model	XBP1 loss in epithelial cells results in intestinal stem cell hyperproliferation	
Tumor quiescence and aggressiveness	Prostate cancer	Change in ATF6*α*, PERK, and IRE1*α* expression	[60, 61, 71, 74, 75]
	B-CLL	BiP/GRP78 overexpression triggers survival signals and prevents apoptosis	
	Triple-negative breast cancers	Constitutively active IRE1*α*/XBP1s axis confers higher aggressiveness due to XBP1-mediated hypoxia-inducible factor-1*α* activation	
	Glioblastoma (GBM)	IRE1*α* endoribonuclease activity regulates the extracellular matrix protein SPARC (secreted protein acidic and rich in cysteine) involved in GBM tumor invasion	
Tumor epithelial-to-mesenchymal transition	Breast tumors thyroid cell glioblastoma (GBM)	Increased expression of XBP1s in metastatic tumors correlates with the EMT inducer SNAIL (snail-related protein) LOXL2 (lysyl oxidase-like 2)/GRP78 activates the IRE1-XBP1 signaling induce EMT-linked transcription factors expression: SNAI1 (snail family transcriptional repressor), SNAI2, ZEB2 (zinc-finger E-box-binding homeobox 2), and TCF3 (transcription factor 3) Serpin B3, a serine/cysteine protease inhibitor overexpression, is associated with chronic UPR induction leading to nuclear factor-*κ*B activation and interleukin-6 production PERK constitutive activation correlates with the overexpression of the TWIST (twist-related protein) transcription factor	[76–78]
Tumor angiogenesis	Human head and neck squamous cell carcinoma	Amino acid deprivation promotes tumor angiogenesis through the GCN2/ATF4 pathway	[32, 63, 65, 79–82]
	Human head and neck squamous cell carcinoma, breast cancer, and glioma cell lines	Glucose deprivation-induced UPR activation promotes upregulation of proangiogenic mediators (VEGF, FGF2, and IL6) and downregulation of several angiogenic inhibitors (THBS1, CXCL14, and CXCL10) through the PERK/ATF4	
	Colorectal cancer	Hypoxic stress-induced PERK overexpression stimulates the creation of microvessels	
	Glioblastoma (GBM)	IRE1*α* signaling induce vascular endothelial growth factor-A (VEGF-A), interleukin-1*β*, and interleukin-6 IRE1*α*-mediated mRNA cleavage of the circadian gene PERIOD1,92 an important mediator of regulation of the CXCL3 chemokine supports tumor angiogenesis PERK-ATF4 branch upregulates VEGF in hypoxia	
	Prostatic and glioma cancer cells	Chaperone ORP150 (oxygen-regulated protein 150) controls tumor angiogenesis by promoting the secretion of VEGF	
Tumor metabolic processes	Triple-negative breast cancer cells	Hypoxia-inducible factor-1*α* activation, XBP1 upregulates glucose transporter 1 expression promotes glucose uptake of IRE1*α*, XBP1s downstream activates enzymes of the hexosamine biosynthetic pathway expression	[83]
Tumor autophagy	Triple-negative breast cancer cells	PERK/eIF2*α*/ATF4 pathway activation protect tumor cells through autophagy induction via LC3B (autophagy protein microtubule-associated protein 1 light chain 3b) and ATG5 (autophagy protein 5) TNF receptor associated factor 2 (TRAF2)/IRE1*α* activates c-Jun N-terminal protein kinase induces autophagy	[19, 83]

**Table 2:** UPR-modulating factors inducing ER stress activity in cancer cells.

Agents	Mechanism	Cancer type/cell lines	References
GSK2606414 and GSK2656157	p-PERK↓, p-elF2*α*↓	Multiple myeloma	[88, 89]
STF-083010	Ire1 inhibitor	Multiple myeloma	[91]
Sorafenib tyrosine kinase inhibitor (TKI)	CHOP↑ GADD34↑; p-PERK↑; p-elF2*α*↑	L-428, L-1236, and KM-H2 cells	[92]
Sal003, inhibitor of phospho-eif2*α* phosphatase	ATF4; p-elF2*α*↑	Glioblastoma cells	[25]
Diindolylmethane derivatives	CHOP↑; DR5↑	Pancreatic cancer cells	[93]
Bortezomib proteasome inhibitor	GRP78↑, CHOP↑, JNK↑, p-eIF2*α*↓	L3.6pl pancreatic cancer cells	[94]
Levistolide A	ROS↑; CHOP↑	Colorectal cancer cells	[97]
Andrographolide	ROS↑; CHOP↑	Colorectal cancer cells	[99]
Tolfenamic acid	eIF2*α*↑; ATF4↑	Colorectal cancer cells	[98]
Cantharidin	GRP78/BiP ↑, IRE1*α* ↑, IRE1β ↑, ATF6*α* ↑, XBP1 ↑	H460	[100]
Carnosic acid	ROS↑; CHOP↑; ATF4↑	Renal carcinoma Caki cells	[101]
Casticin	CHOP ↑, p-eIF2*α* ↑, eIF2*α* ↑, GRP78/BiP ↑	BGC-823	[102]
Cryptotanshinone	p-eIF2*α* ↑, GRP94 ↑, GRP78 ↑, CHOP ↑, ROS↑	MCF7	[103]
Curcumin	CHOP ↑, GRP78/BiP ↑, ROS ↑	NCI-H460, HT-29, AGS	[104, 105]
Flavokawain B	CHOP ↑, ATF4 ↑	HCT116	[106]
Fucoidan	CHOP ↑, ATF4 ↑, p-eIF2*α* ↑, GRP78/BiP ↓, p-IRE1 ↓, XBP1 ↓	MDA-MB-231 HCT116	[107]
Furanodiene	CHOP ↑, BIP ↑	A549, 95-D	[108]
2-3,4 Dihydroxyphenylethanol	IRE1 ↑, XBP1 ↑, GRP78/BiP ↑, PERK ↑, eIF2*α* ↑, CHOP ↑	HT-29	[109]
7-Dimethoxyflavone	CHOP ↑, GPR78/BiP ↑, ATF4 ↑	Hep3B	[110]
SMIP004 (N-(4-butyl-2-methyl-phenyl) acetamide)	ROS↑ IRE1↑; p-38↑; p-elF2*α*↑	Prostate cancer cells	[111]
Licochalcone A	ATF6 ↑, eIF2*α* ↑, IRE1*α* ↑, CHOP ↑, GRP94 ↑, XBP1 ↑, GRP78/BiP ↑	HepG2	[112]
Neferine	GRP78/BiP ↑	Hep3B	[113]
Paeonol	GRP78 ↑, CHOP ↑	HepG2	[114]
Pardaxin	ROS↑; p-PERK↑; p-elF2*α*↑	HeLa cells	[115]
Parthenolide	ATF4 ↑, p-eIF2a ↑, eIF2*α* ↑	A549, Calu-1, H1299, H1792	[116]
Piperine	IRE1*α* ↑, CHOP ↑, GPR78/BiP ↑	HT-29	[117]
Polyphenon E	ATF4 ↑, PERK ↑, p-eIF2*α* ↑, eIF2*α* ↑, GRP78/BiP ↑, CHOP ↑, XBP1 ↑, ROS ↑	PC3, PNT1a	[118]
Polyphyllin D	CHOP ↑, GRP78/BiP ↑, PDI ↑	NCI-H460	[119]
Resveratrol	GRP78/BiP ↑, CHOP ↑, XBP1 ↑, eIF2*α* ↑	HT29	[120]
Dehydrocostuslactone	p-PERK ↑, GRP78/BiP ↑, IRE1 ↑, CHOP ↑, XBP-1 ↑, ROS ↑	NCI-H460 A549	[121]
*γ*-Tocotrienol	CHOP ↑, GRP78/BiP ↑, XBP1 ↑	MDA-MB-231; MCF-7	[122]
Ω-Hydroxyundec-9-enoic Acid (*ω*-HUA)	ROS↑; CHOP↑	Lung cancer cells (H1299, A549, HCC827)	[123]
Ampelopsin	ROS↑ GRP78↑; p-PERK↑; p-elF2*α*↑	Breast cancer cells (MCF-7; MDA-MB-231)	[124]
Ardisianone	GRP78/BiP ↑	PC3	[125]
Genistein	CHOP ↑, GRP78/BiP ↑	Hep3B	[126]
Guttiferone H	ATF4 ↑, XBP1 ↑, CHOP ↑	HCT116	[127]
Guggulsterone	ROS↑; p-eIF2*α*↑; CHOP↑ DR5↑	Liver cancer cells (Hep3B; HepG2)	[128]
Marchantin M	GRP78/BiP, CHOP ↑, XBP1 ↑, p-eIF2*α* ↑, eIF2*α* ↑, ATF4 ↑, ATF6 ↑, ERAD ↓	PC3, DU145, LNCaP	[129]
Sarsasapogenin	ROS↑; CHOP↑	HeLa cells	[130]
Saxifragifolin	IRE1*α* ↑, XBP1 ↑, CHOP ↑, GRP78/BiP ↑, ROS↑	MDA-MB-231, MCF7	[131]
Prodigiosin	ROS↑; CHOP↑; p-eIF2*α*↑; PERK↑; GRP78↑; ATF6*α*↑, IRE1 ↑, eIF2a ↑	Pancreatic (8898); breast cancer cells (MCF-7 and MDA-MB-231)	[132, 133]
Quercetin	GRP78/BiP ↑, ATF4 ↑, IRE1*α* ↑ ATF6 ↑	PC3	[134]
Honokiol (HNK)	ROS↑ p-eIF2*α*↑; GRP78↑ CHOP↑	Chondrosarcoma (JJ012 and SW1353); gastric (AGS, SCM-1 and MKN-45) cancer cells	[135–139]
Brefeldin A (BFA)	ROS↑, IRE1*α* ↑, PERK ↑, XBP1↑; GRP78↑ CHOP↑	Ovarian (OVCAR-3); lung (A549); colorectal (colo 205); breast (MDA-MB-231) cancer cells	[140–142]
A-tocopheryl succinate	SGC-7901	GRP78/BiP ↑, CHOP ↑	[143]
Verrucarin A	GRP78/BiP ↑, p-PERK ↑, p-eIF2*α* ↑, CHOP ↑	Hep3B, HepG2	[144]
Vitamin E succinate	GRP78/BiP ↑, GRP94 ↓, PERK ↑, ATF4 ↑, ATF6 ↑, XBP1 ↑, CHOP ↑	SGC-7901	[145]
Ultrafine	p-eIF2*α* ↑, GRP78/BiP ↑	SNU-484	[146]
Zerumbone	ATF4 ↑, CHOP ↑, GRP78/BiP ↑, p-PERK ↑, PERK ↑ eIF2*α* ↑, p-eIF2*α* ↑	HCT116-p53null, SW480, PC3	[68, 147]
